# Interaction of a Novel Zn2Cys6 Transcription Factor DcGliZ with Promoters in the Gliotoxin Biosynthetic Gene Cluster of the Deep-Sea-Derived Fungus *Dichotomomyces cejpii*

**DOI:** 10.3390/biom10010056

**Published:** 2019-12-29

**Authors:** Zi-Lei Huang, Wei Ye, Mu-Zi Zhu, Ya-Li Kong, Sai-Ni Li, Shan Liu, Wei-Min Zhang

**Affiliations:** State Key Laboratory of Applied Microbiology Southern China, Guangdong Provincial Key Laboratory of Microbial Culture Collection and Application, Guangdong Open Laboratory of Applied Microbiology, Guangdong Institute of Microbiology, Guangdong Academy of Sciences, Guangzhou 510070, China; huangzilei15@mails.ucas.ac.cn (Z.-L.H.); zhumz@gdim.cn (M.-Z.Z.); 13610201546@163.com (Y.-L.K.); lisn@gdim.cn (S.-N.L.); 2014168125@ctgu.edu.cn (S.L.)

**Keywords:** *Dichotomomyces cejpii*, DcGliZ regulator, gliotoxin biosynthesis, electrophoretic mobility shift assay, surface plasmon resonance

## Abstract

Gliotoxin is an important epipolythiodioxopiperazine, which was biosynthesized by the *gli* gene cluster in *Aspergillus genus*. However, the regulatory mechanism of gliotoxin biosynthesis remains unclear. In this study, a novel Zn2Cys6 transcription factor DcGliZ that is responsible for the regulation of gliotoxin biosynthesis from the deep-sea-derived fungus *Dichotomomyces cejpii* was identified. DcGliZ was expressed in *Escherichia coli* and effectively purified from inclusion bodies by refolding. Using electrophoretic mobility shift assay, we demonstrated that purified DcGliZ can bind to g*liG*, *gliM*, and *gliN* promoter regions in the *gli* cluster. Furthermore, the binding kinetics and affinity of DcGliZ protein with different promoters were measured by surface plasmon resonance assays, and the results demonstrated the significant interaction of DcGliZ with the *gliG, gliM,* and *gliN* promoters. These new findings would lay the foundation for the elucidation of future gliotoxin biosynthetic regulation mechanisms in *D. cejpii*.

## 1. Introduction

Marine-derived fungi can produce diverse natural products with different bioactives and have become a more and more important resource of leading compounds over the past years [1]. Diketopiperazine derivatives show a wide range of biological activities, including antiviral, antituberculosis, anticancer, and antibacterial activities [2,3]. Plinabulin, a synthetic diketopiperazine analogue derived from marine fungus *Aspergillus* sp. CNC-139, has entered phase III clinical studies for the treatment of non-small cell lung cancer, indicating the bright prospect of gliotoxin derivatives in drug development [4,5,6]. Gliotoxin, derived from fungi, is a typical epipolythiodioxopiperazine (ETP) toxin with a disulphide-bridged cyclic dipeptide, and is responsible for the cytotoxicity [7,8]. Moreover, gliotoxins and their derivates can utilize the disulfide bond of ETP compounds to mediate redox homeostasis or the modification of proteins, including nuclear factor-kB, triphosphopyridine nucleotide (NADPH) oxidase, histone H3 lysine 9 (H3K9) methyltransferase, and glutaredoxin [9]. In addition to the diverse extraordinary structures and bioactivities of gliotoxins, the biosynthesis pathways of gliotoxins and relevant genes have been identified in *Aspergillus fumigatus*, which would provide theoretical guidance for multiple functional genomic studies in other fungi [8,9].

The putative gene cluster (*gli*) of gliotoxin is composed of 13 genes that encode crucial enzymes responsible for the biosynthesis of various gliotoxins and their derivatives in *A. fumigatus* [9]. The non-ribosomal peptide synthetase encoded by *gliP* gene catalyzes diketopiperazine scaffold formation, the first biosynthetic reaction in a gliotoxin biosynthesis pathway [10]. Subsequently, cytochrome P450 monooxygenase encoded by *gliC* catalyzes the hydroxylation at the α-carbon of L-Phe [11]. Then, glutathione S-transferase (GST) encoded by the *gliG* promotes the sulfurization of gliotoxin biosynthetic imtermediates; GST is known for its ability in the catalyzation of carbon–sulfur bond formation, as opposed to detoxification [12,13]. After the process by the enzymes of GliK and GliJ, the γ-glutamyl moieties are removed [14,15]. The carbon–sulfur lyase expressed by the *gliI* gene then catalyzes the intermediate to generate a notorious epidithiol moiety [16]. After the catalysis of cytochrome P450 monooxygenases (GliF or GliC) and GliH (function remains elusive), the N-methyltransferase encoded by *gliN* or *gliM* functions as a freestanding amide to promote amide methylation and confer stability on ETP [17,18]. Finally, the oxidoreductase GliT-mediated disulfide bridge closure might be a prerequisite for the formation of gliotoxins, and the major facilitator superfamily transporter GliA also plays an important role in exporting the toxins to prevent the harmful effect of gliotoxin on hosts [9,19].

Many regulators involved in gliotoxin biosynthesis pathway have been identified in *A. fumigatus*, regardless of whether they deploy a non-*gli* cluster or a *gli* cluster. GtmA (or termed TtmA) is encoded outside the *gli* cluster and functions as a bis-thiomethyl transferase for the conversion of dithiogliotoxin to bisdethiobis (methylthio) gliotoxin (BmGT), which mainly attenuates the formation of disulfide bridge closure [20]. Other non-*gli* clusters encoding transcription factors, including the global regulator laeA, C_2_H_2_ regulator mtfA, bZIP transcription factor rsmA, and APSES family transcription factor stuA, can regulate the biosynthesis of gliotoxins and their derivatives to some extent [9]. The critical transcription factor GliZ encoded by the *gliZ* gene in the *gli* cluster is a sequence-specific DNA-binding binuclear zinc cluster (Zn_2_Cys_6_) protein, which is uniquely found in fungi. GliZ is a positive regulator that is indispensable in the gliotoxin biosynthesis [21]. Moreover, the overexpression of the *gliZ* gene leads to the accumulation of gliotoxins and their derivatives, and the *gliZ* deletion impedes the production of gliotoxins. The binding site (TCGGN3CCGA) of GliZ is usually located within the promoter region of the *gli* cluster [7,21,22,23]. Although the regulatory function of *gliZ* has been preliminarily demonstrated through in vivo experiments, the specific promoter and the evaluation of binding affinities between *gli* gene promoters and GliZ remain obscure. Therefore, it is of great significance to elucidate the regulatory mechanism of GliZ by in vitro analysis of the interaction of GliZ with promoters in the gliotoxin biosynthetic cluster in different fungi.

Different kinds of gliotoxins and their derivatives including rare gliotoxin dimers were isolated from *Dichotomomyces cejpii* (Milko) Scott in our previous study, and some showed significant cytotoxic activities against tumor cell lines and high inhibitory activity against α-glucosidase [24,25,26], which exhibited the potential to be developed as leading compounds for drugs in the future. However, the novel transcriptional factor GliZ in the *gli* cluster responsible for the gliotoxin biosynthesis in *D. cejpii* (DcGliZ) remains unclear, which is unfavorable for the elucidation of the biosynthetic and regulatory mechanism of gliotoxins and their derivatives in *D. cejpii.* Thus, it is urgent to investigate the regulatory function of this transcriptional factor GliZ in *D. cejpii*. To explore the specific regulatory mechanism of GliZ for the gliotoxins biosynthetic genes in the *gli* cluster, thus facilitating the biosynthesis of new gliotoxin derivatives in the future, an effective approach for identifying the function and regulatory mechanism of DcGliZ (accession number: MK558823) was proposed in this study. Using the whole genome sequencing data of the deep-sea-derived fungus *D. cejpii* FS110, the available gliotoxin synthetic gene cluster and its relevant transcriptional factor DcGliZ were predicted. Moreover, the binding abilities of DcGliZ to promoters of genes in the *gli* cluster was assayed by electrophoretic mobility shift assay (EMSA) and surface plasmon resonance (SPR) assays. This is the first report on the investigation of the interaction between DcGliZ and the promoters of gliotoxins biosynthetic genes in the *gli* cluster in vitro, and the disclosure of the binding levels between transcriptional factor DcGliZ and *gliG*, *gliM*, and *gliN* promoters by measuring the equilibrium dissociation constant (K_D_). Our study would promote the elucidation of the gliotoxin biosynthetic mechanism, and provide a platform for the precise regulation of the *gli* cluster to control gliotoxin production and excavate new gliotoxin derivatives through the regulation of DcGliZ expression and the mutation of promoters for gliotoxins biosynthesis.

## 2. Materials and Methods

### 2.1. Genome Sequencing and Assembly

The strain *D. cejpii* FS110 (Accession No.KF706672) was isolated from a deep-sea sedimental sample in the South China Sea, and single colonies were incubated on a potato dextrose agar medium for five days at 30 °C [27]. The total genome DNA of *D. cejpii* FS110 was extracted using a Charge Switch gDNA Mini Bacteria Kit (Life Technologies, Carlsbad, CA, USA) according to the manufacturer’s protocol. Genome DNA was checked using TBS-380 (Turner Biosystem Inc, Sunnyvale, CA, USA) for the production of high-quality dsDNA (OD260/280 = 1.8–2.0, >10 μg) and subsequent sequence. For the construction of a genome library, the purified genome DNA was broken into short fragments, which were used for Illumina sequencing (Illumina HiSeq Xten, San Diego, CA, USA) and Pacific Biosciences sequencing (Pacific Biosciences RS, San Diego, CA, USA), according to the manufacturer’s instructions. The fragments, including the Illumina and PacBio reads, were assembled by SOAP denovo v2.04 into many long scaffolds and finally integrated into a complete genome sequence [28]. This whole genome shotgun project of *D. cejpii* FS110 was deposited at DDBJ/ENA/GenBank under the accession SMSW00000000.

### 2.2. Gli Cluster Prediction and Annotation

Gene prediction was achieved through MAKER2 process including a variety of prediction methods, such as SNAP, AUGUSTUS version 2.5.5 (Greifswald, Germay) and GeneMark-ES version 2.3a (Atlanta, Georgia, USA) [29,30]. The predicted genes were annotated on the basis of protein sequence alignments obtained with different databases, including NR, Swiss-prot, Pfam, GO, COG, and KEGG databases, all of which provided detailed information on protein function analysis. Annotated genomes were submitted to antiSMASH for the detection of the gliotoxin secondary metabolic gene cluster (*gli*) of *D*. *cejpii* FS110, and all possible genes related to gliotoxin biosynthesis were annotated by comparing with the *A. fumigatus gli* cluster [31]. The promoters of the *gli* cluster genes were predicted using PromPredict.

### 2.3. Conserved Domain Prediction and Phylogenetic Analysis of DcGliZ

The coding sequence of transcriptional factor DcGliZ in *D*. *cejpii* FS110 was predicted by open reading frame (ORF) finder (http://www.ncbi.nlm.nih.gov/gorf/gorf.html) on the basis of the genome sequence, and the protein sequence of DcGliZ was obtained with Snap gene 2.3.2. The conserved domain Zn_2_Cys_6_ of DcGliZ was identified on the Pfam server, and the 3D structure and DNA binding region of Zn_2_Cys_6_ were illustrated by SWISS-MODEL and PyMol [32].

To analyze the protein phylogeny between DcGliZ and another GliZ type of transcriptional factors, six fungal GliZ orthologues relevant to gliotoxin ETP biosynthesis were selected from the following species for sequence alignment and phylogenetic tree construction using the neighbor joining method of Geneious program: *Aspergillus oryzae* (Swiss-Prot: Q2UPC3.1, AclZ), *Aspergillus flavus* (GenBank: RAQ76477.1, AflaGliZ), *Leptosphaeria maculans* (GenBank: AAS92551.1, SirZ), *Aspergillus fumigatus* (GenBank: AAW03310.1, AfGliZ), *Penicillium lilacinoechinulatum* (GenBank: ABV48732.1, PlGliZ), and *Trichoderma virens* (GenBank: ABV48713.1, TvGliZ); and four fungal GliZs unrelated to gliotoxin synthesis from *Metarhizium brunneum* (NCBI Reference Sequence: XP_014543367.1, MbGliZ), *Rasamsonia emersonii* (NCBI Reference Sequence: XP_013327000.1, ReGliZ), *Talaromyces stipitatus* (GenBank: EED16355.1, TsGliZ), and *Talaromyces-marneffei* (GenBank: EEA20409.1, TmGliZ) [21,23,33,34,35]. The conserved domain and phylogenetic relationship of these GliZ transcriptional factors were clearly identified.

### 2.4. Heterologous Expression and Purification of Full-Length DcGliZ and DcGliZ Core Proteins from E. coli

The total RNA of *D*. *cejpii* FS110 was extracted using an RNA extracting kit (Magen, Shanghai, China), and the first-stand cDNA was synthesized using a reverse transcription kit (Abm, Vancouver, Canada). The full-length DcGliZ gene, DcGliZ core gene, and vector pET22b were amplified by the primers listed in Table S1, and then the recombinant vectors of full-length pET22b-DcGliZ and pET22b-DcGliZ (core) were constructed through circular polymerase extension cloning (CPEC) [36]. The recombinant vectors pET22b-DcGliZ (full length) and pET22b-DcGliZ (core) were induced using 1 M of IPTG for 4 h at 37 °C and for 6 h at 22 °C, respectively, and then expressed in 200 mL of *Escherichia coli* BL21 (DE3). The fermentation liquids containing pET22b-DcGliZ full-length and pET22b-DcGliZ core vectors were centrifuged for the collection of pellet at 4 °C (8000× *g*, 5 min) and resuspended in 20 mL of 10 mM HEPES (pH 7.4). Then, 20 mL of the resuspended pellet was ultrasonicated with 35% amplitude for 60 min and centrifuged at 4 °C (12,000× *g*, 15 min). The DcGliZ core protein was dissolved in the supernatant and the full-length DcGliZ protein was present in inclusion bodies.

DcGliZ core protein (10 mL) was added to an Ni-affinity chromatography column and washed twice with 10 mM HEPES (pH 7.4), and then the column was eluted with 10 mM HEPES (pH 7.4) containing different concentrations of imidazole (50, 150, and 500 mM). Different DcGliZ core protein samples were collected with a tube, and then 40 μL of each extracted sample was added to 10 μL of sodium dodecyl sulfate (SDS)-loading buffer and incubated for 5 min in boiled water. Different protein samples were identified by sodium dodecyl sulfate-polyacrylamide gel electrophoresis (SDS-PAGE) after coomassie blue staining. Finally, the eluted target protein was deionized using 10 kDa ultrafiltration (Millipore, Massachusetts, Germany) for further study.

Owing to the existence of full-length DcGliZ proteins as inclusion bodies, an effective refolding protein strategy was developed. Full-length DcGliZ inclusion bodies were solubilized in 10 mM HEPES containing 6 M guanidine hydrochloride (GH, pH 8.5) and centrifuged at 4 °C (12,000× *g*, 5 min). Then, the supernatant was transferred to a new tube. The dissolved full-length DcGliZ protein was dialyzed for renaturation in a 40 kDa dialysis tube with a refolding buffer (0.4 M L-Arg, 10 mM HEPES, 2 mM GSH, 0.4 mM GSSG, and 0.15 M NaCl) with gradient concentrations of GH (4, 2, 1, 0.5, 0 M) at 4 °C for 12 h. Finally, the full-length DcGliZ protein was dialyzed in 10 mM HEPES (pH 4.5) and filtrated by 0.22 μm millipore filter, and the concentration of the full-length DcGliZ protein was measured with a spectrophotometer (NanoDrop Life, Massachusetts, USA) and stored at 4 °C for further use.

### 2.5. Interaction Between Transcriptional Factor DcGliZ and the Promoters of the Gli Cluster

The promoters of genes relevant to gliotoxin biosynthesis in the *gli* cluster were cloned using the primers listed in Table S2 and purified with a Hipure gel pure DNA kit (Magen, Shanghai, China). The purified promoters, including *pG*, *pM*, *pCP*, *pI*, *pTF*, *pN*, and *pA,* were used for the production of a biotin probe. An EMSA probe biotin labeling kit (Beyotime, Shanghai, China) was used. Then, 5 μL of each promoter-biotin probe (1 μM) was mixed with 2 μL full-length DcGliZ transcriptional factor (about 1 mg/mL) for EMSA using the Chemiluminescent EMSA Kit (Beyotime, Shanghai, China), respectively. After the promoters that can combine with the full-length DcGliZ were determined, the 2 μL DcGliZ (core) protein (about 1 mg/mL) was incubated with the promoters for EMSA for the identification of the functional domain of DcGliZ. The *pG* promoter was divided into three parts (G1-1, G1-2, and G1-3 (1 μM), shown in Supplementary Materials) to ensure which region could bind to the core protein of DcGliZ.

### 2.6. Surface Plasmon Resonance Assay

SPR assays were performed in Biacore T200 biosensor (GE Healthcare Life Sciences, Uppsala, Sweden) with a CM5 sensor chip. All the experiments were carried out at a constant temperature of 22 °C using HBS-EP (10 mM HEPES pH 7.4, 150 mM NaCl, and 0.005% (*v*/*v*) detergent P20) as running buffer [35,36,37,38]. Before the SPR assays, the ligand coupling amount was calculated by the following formula (R_L_: ligand coupling value, S_m_: stoichio–metric ratio, R_max_: maximal binding unit of CM5 chip, ligand/analyte MW: molecular weight), and the actual value was 1.5 R_L_.

(1) $$\mathrm{RL}=\frac{\mathrm{Rmax}\times \mathrm{ligandMW}}{\mathrm{Sm}\times \mathrm{analyteMW}}$$

The purified full-length DcGliZ protein was diluted to 20 μg/mL by sodium acetate at different pH levels (4.0, 4.5, 5.0, and 5.5) for pH scouting and the identification of the optimal immobilization condition. Then, the CM5 chip was activated by injecting a 1:1 ratio of 0.1 M nhydroxysuccinimide and 0.4 M N-(3-dimethylaminopropyl)-N′-ethylcarbodiimide hydro-chloride, and the diluted full length DcGliZ was immobilized on the surface of the CM5 chip. The remaining activated group on the chip surface was blocked with 0.5 M ethanolamine for about 3 min [39]. The immobilization level (response unit, RU) of the full-length DcGliZ protein can be calculated by a Biacore instrument.

The SPR analysis was carried out using different concentrations of promoters to validate whether the *gli* promoters can combine with full-length DcGliZ, and the contact time was set to 60 s (*pG, pM, pN* gradient concentration: 1024, 512, 256, 128, 64, 32, 16, and 0 nM. *pCP* gradient concentration: 256, 128, 64, 32, 16, 8, 4, 4, and 0 nM). Furthermore, the bioactivity and amount of full-length DcGliZ protein on the chip surface after the multicycle test was observed, and a reasonable regeneration scouting assay was necessary for the screening of affinity and kinetics. Thus, 10 mM glycine-HCl at different pH levels (1.5, 2.0, and 2.5) was used for the screening of regeneration conditions. The kinetics experiments were implemented by injecting different promoters of the *gli* cluster with different concentrations at a flow rate of 30 μL/min, and the contact time was set to 120 s. Dissociation time was set to 60 s. The response signal was captured by the sensing system. Finally, the affinity and kinetics data were analyzed with Biacore T200 (GE Health Care, Freiburg, Germany).

## 3. Results

### 3.1. Prediction and Analysis of the Gliotoxin Biosynthesis Cluster of D. cejpii

**Figure 1:**
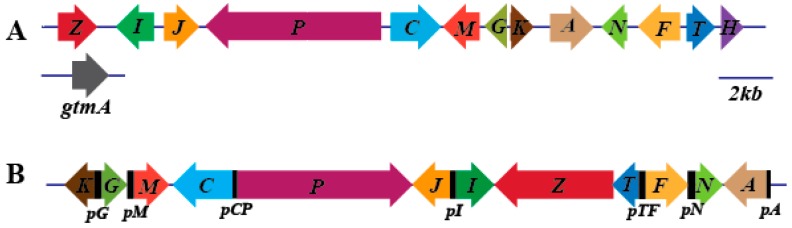
Gliotoxin biosynthesis gene (*gli*) cluster. (**A**) *gli* cluster region contains 13 genes located on chromosome 6, and the *gtmA* gene is located on chromosome 3, all these genes are derived from *A*. *fumigatus*. (**B**) Putative *gli* cluster, which encodes the crucial enzyme for the biosynthesis of gliotoxins from *D*. *cejpii* (all functional genes in color were labeled with their capital letters, black square represents gene promoters of *gli* cluster, *pG*, *pM*, *pCP*, *pI*, *pTF*, *pN,* and *pA*).

The genome of *D. cejpii* was sequenced, assembled, and annotated. Approximately 8065 genes were predicted, and the length of the total genome sequence was 23,887,422 nt. The gene clusters for the biosynthesis of secondary metabolites were predicted using antiSMASH version 5.0.0. Seventeen gene clusters comprising gli cluster for gliotoxin biosynthesis were found in the genome of *D. cejpii* (Figure S1). A total of 12 putative gliotoxin synthetic genes (approximately 27.5 kb) were predicted, and the functional annotation of the genes using eukaryotic orthologous groups indicated that almost all genes in the *gli* cluster of *D. cejpii* can be found in the *gli* cluster in *A. fumigatus*, suggesting the similar function of these genes including *gliZ* as gliotxin biosynthetic genes (Figure 1). However, the difference in the genes’ arrangements between the *gli* clusters of *D. cejpii* and A. fumigatus suggested the different biosynthetic mechanisms of gliotoxins for the two fungi. Furthermore, the *gliM* gene was annotated as an O-methyltransferase by gene prediction, and the *gliH* gene (function remains unclear) was related to the synthesis of gliotoxin lacking in *D. cejpii*, indicating that different chemical modification by the enzymatic reaction might exist. The promoters of genes in the *gli* cluster of *D. cejpii* were predicted and named as *pG, pM, pCP, pI, pTF, pN,* and *pA*, respectively.

### 3.2. Conserved Domain of Zn2Cys6 Transcriptional Factor DcGliZ

The complete genome sequence of *D*. *cejpii* enabled the robust prediction of the *gli* cluster transcriptional factor DcGliZ. The Zn_2_Cys_6_ domain of DcGliZ was located in the region between amino acids at the positions of 16 and 59 according to Pfam analysis, and the 3D model of partial sequence of DcGliZ (mainly Zn_2_Cys_6_ region) was created using SWISS-MODEL and PyMol (Fig.S2A). The model illustrated that the structure of Zn_2_Cys_6_ contained an α-helix and two β-sheets, and the amino acid in DcGliZ including cys22, cys25, cys32, cys38, cys41, and cys48 were covalently linked with two zinc ions, which facilitated the formation and stabilization of the finger configuration, and thus ensured the regulatory function of DcGliZ by completely inlaying the major groove of the specific DNA sequence.

**Figure 2:**
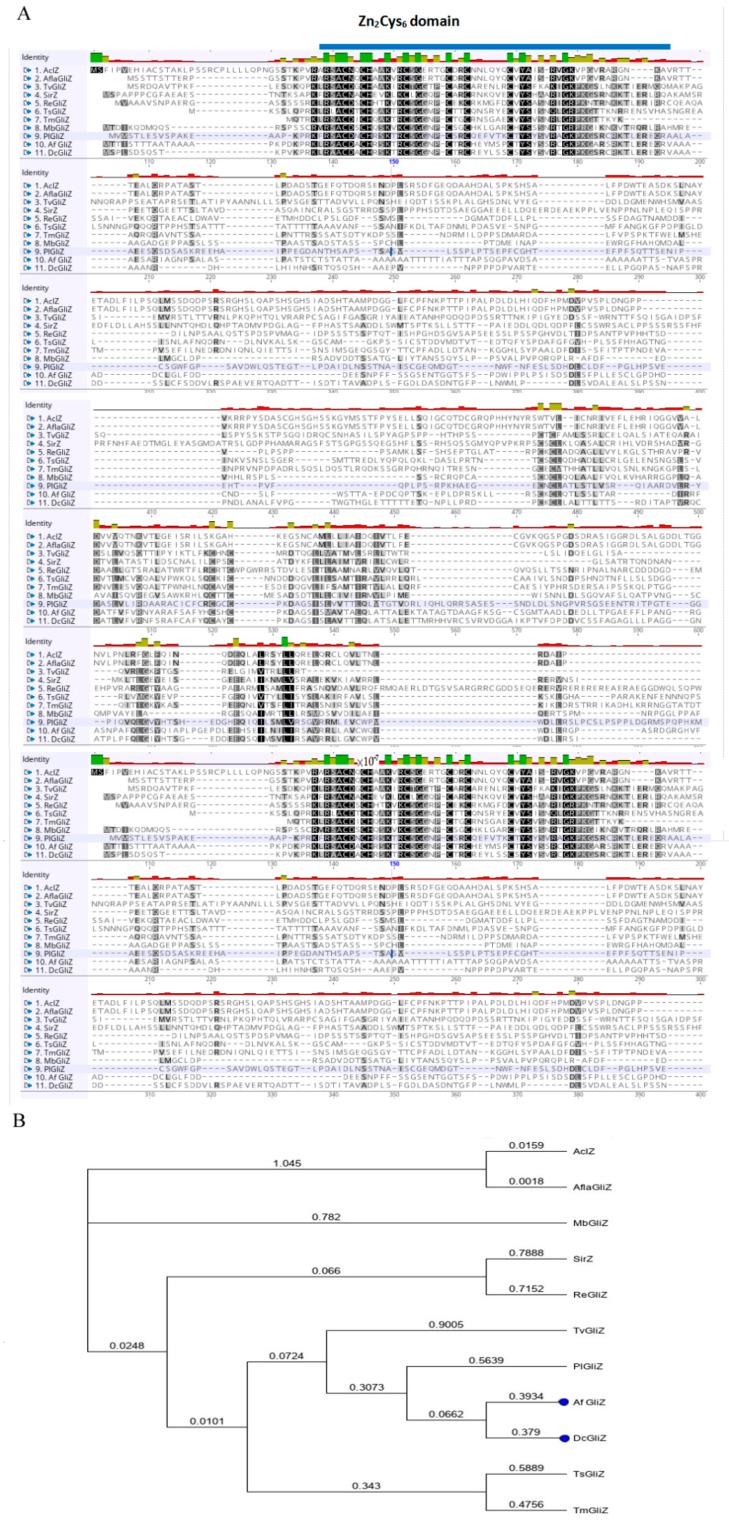
Phylogenetic analysis of DcGliZ transcription factor. (**A**) Alignment of DcGliZ with other types of GliZs from 10 different species of fungi. Seven fungal GliZs related to gliotoxin synthesis: *Aspergillus oryzae* (AclZ); *Aspergillus flavus* (AflaGliZ), *Leptosphaeria maculans* (SirGliZ), *Aspergillus fumigatus* (AfGliZ), *Penicillium lilacinoechinulatum* (PlGliZ), *Dichotomomyces cejpii* (DcGliZ), and *Trichoderma virens* (TvGliZ); and four fungal GliZs unrelated to gliotoxin synthesis: *Metarhizium brunneum* (MbZn_2_Cys_6_), *Rasamsonia emersonii* (ReZn_2_Cys_6_), *Talaromyces stipitatus* (TsGliZ), and *Talaromyces marneffei* (TmZn_2_Cys_6_). The blue line denotes the putative Zn_2_Cys_6_ domain, black shade indicates almost identical amino acids, and gray shade indicates more similar amino acids. (**B**) Phylogenetic tree construction of full sequences from eleven ZnCys_6_ -type GliZs using the Geneious program (the value 0.2 means genetic distance).

For the determination of the phylogenetic relationship of DcGliZ with other Zn_2_Cys_6_-type GliZs derived from different fungi, alignment analysis was carried out. The similarity between DcGliZ and AclZ, AflaGliZ, SirGliZ, AfGliZ, PlGliZ, and TvGliZ was 37.5%, 38.5%, 38.5%, 53.2%, 48.2%, and 35.4%, respectively. The results indicated that the DcGliZ sequence of *D*. *cejpii* showed high similarity to other fungal GliZs in the DNA binding region Zn_2_Cys_6_ (Figure 2A), suggesting that the regulatory mechanism for gliotoxin biosynthesis in *D*. *cejpii* is similar to that in other fungi. Moreover, the phylogenetic tree constructed using the Geneious program indicated that the DcGliZ showed the highest genetic relationship with that of *A. fumigatus* GliZ (Figure 2B). The similarity of the two GliZs was 38.5%, implying the similar function of DcGliZ as a positive regulator for the gliotoxin biosynthesis.

### 3.3. Heterologous Expression and Purification of Full-Length DcGliZ and DcGliZ Core Proteins from E. coli

**Figure 3:**
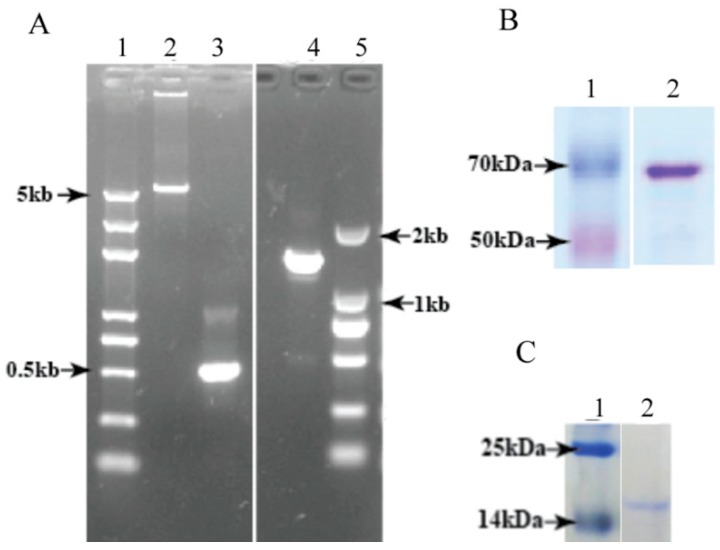
Expression, purification, and renaturation of full-length DcGliZ and DcGliZ core proteins. (**A**) Amplification of full-length *gliZ* and *gliZ* core genes from *D. cejpii* by CPEC PCR: lane 1, 5 kb size marker; lane 2, pET22B vector PCR products; lane 3, *gliZ* core gene PCR products; lane 4, full-length *gliZ* gene PCR products; lane 5, 2 kb size marker. (**B)** Sodium dodecyl sulfate-polyacrylamide gel electrophoresis (SDS-PAGE) of full-length DcGliZ protein after Coomassie blue staining: lane 1, 70 kDa size marker; lane 2, 10 μL refolded and purified full-length DcGliZ protein (1 mg/mL). (**C**) SDS-PAGE of DcGliZ core protein after Coomassie blue staining: lane 1, 25 kDa size marker; lane 2, purified DcGliZ core protein.

To verify the function of the full-length DcGliZ and the Zn_2_Cys_6_ domain of DcGliZ, the plasmids containing the full-length DcGliZ and core DcGliZ were constructed (Figure 3A); one was the pET22b-core region of DcGliZ expression system containing the first 160 amino acid sequences of full-length DcGliZ, which mainly consisted of the Zn_2_Cys_6_ domain (Molecular weight-17.45 kDa, pI-8.6; Figure 3B), and the other was the pET22B-DcGliZ full-length expression system containing the full DcGliZ protein sequence (Molecular weight-50 kDa, pI-6.46; Figure 3C). The DcGliZ core protein expressed in *E. coli* was soluble, whereas the full-length DcGliZ protein expressed in *E. coli* was insoluble inclusion bodies (IBs). Thus, a more effective strategy was proposed for the recovery of the misfolded full-length DcGliZ protein and retention of its bioactivity. The results indicated that full-length DcGliZ protein was successfully recovered and purified from IBs and the DcGliZ core protein was also purified from the supernatant of *E. coli* containing recombinant plasmid after sonication. Finally, we got approximately 5 mL full-length DcGliZ protein (100 mg/mL) and 3 mL DcGliZ core protein (100 mg/mL).

### 3.4. Electrophoretic Mobility Shift Assay between Full-Length DcGliZ or DcGliZ Core Protein and Functional Gene Promoters of the Gli Cluster

**Figure 4:**
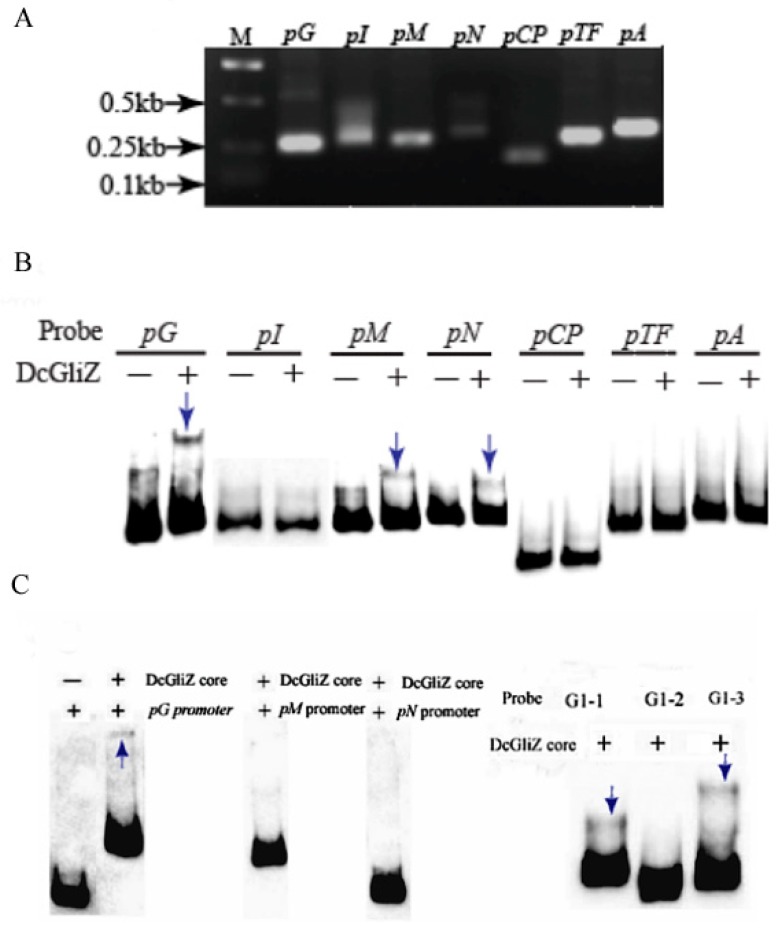
Schematic presentation of the interaction between full-length or core DcGliZ and promoters. (**A**) Clone of promoters of the *gli* cluster genes (1 μM). (**B**) Gel retardation assay of 2 μL full-length DcGliZ (100 mg/mL) with 5 μL of each probe (1 μM). (**C**) Gel retardation of 2 μL DcGliZ core (100 mg/mL) with 5 μL of each probe (1 μM). (‘-’means with no sample, ‘+’means adding 2 μL sample, and ‘blue arrow’ shows gel retardation; the concentrations of DcGliZ core and full-length DcGliZ protein are both approximately 100 mg/mL).

To determine which gene promoter within the *gli* cluster can combine with the full-length DcGliZ transcriptional factor in *D*. *cejpii*, *pG*, *pM*, *pCP*, *pI*, *pTF*, *pN*, and *pA* promoters for gliotoxins biosynthesis were cloned and their corresponding probes were prepared (Figure 4A). The in vitro EMSA experiment results indicated that the full-length DcGliZ protein can only bind to *pG*, *pM*, and *pN* promoters, and the retardation band of the *pG* promoter probe with DcGliZ was more clear compared with those of the other two promoters (Figure 4B). The sequences of promoters *pG, pM,* and *pN* were labeled with biotin, and then the DcGliZ core protein was added to finish the EMSA assay. The retardation band was observed in DcGliZ core protein–*pG* complex, however, no such retardation band was observed in DcGliZ core protein–*pM* complex and DcGliZ core protein–*pN* complex. The sequence of *pG* promoter was divided into three segments of equal length (G1-1, G1-2, and G1-3) and retardation bands were observed in DcGliZ core protein–G1-1 complex and DcGliZ core protein G1-3 (Figure 4C). The results indicated that, although the core Zn_2_Cys_6_ domain of DcGliZ plays a dominant role in the regulation of gliotoxin biosynthesis, other domains or structures in DcGliZ may exist for some unknown regulatory mechanism, owing to the non-binding DcGliZ core protein–*pM* complex and DcGliZ core protein–*pN* complex.

### 3.5. Affinity and Kinetics Analysis between DcGliZ and Gli Cluster Gene Promoters

To acquire the accurate binding affinity between full-length DcGliZ and promoters for gliotoxins biosynthesis, the commercial Biacore T200 SPR instrument was employed to a achieve highly specific and sensitive analysis. The factual ligand (full-length DcGliZ) coupling amount (RL) measured by Biacore T200 was 325. The optimal pH condition of 10 mM sodium acetate for the full-length DcGliZ immobilization on the surface of the CM5 chip was 3.0, which was confirmed by the pH scouting experiments, and adequate ligand for promoter capture was provided. Then, glycine-HCl buffer with an optimal pH value of 2.0 for the surface test and regeneration to scout four different promoters guaranteed the accuracy of the affinity and kinetic data.

**Figure 5:**
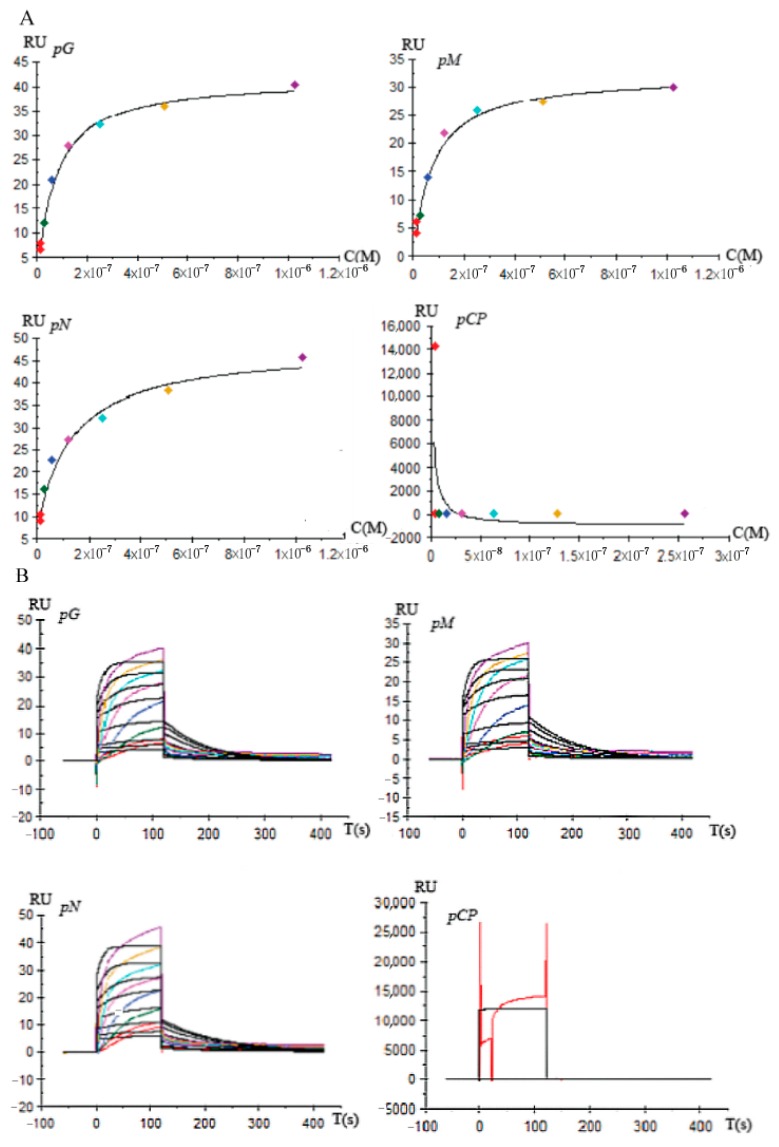
Affinity and kinetics test identified the precise interaction between full-length DcGliZ and *pG*, *pM, pN*, and *pCP* promoters. (**A**) Dynamic variation of affinity curve between DcGliZ and *pG*, *pM, pN*, and *pCP* promoters (vertical axis represents response unit (RU), horizontal axis represents molar concentration of promoter (M)). (**B**) Dynamic change of kinetics fitting curve between full-length DcGliZ and *pG*, *pM, pN*, and *pCP* promoters (vertical axis represents response value (RU), horizontal axis represents combining time (s)).

The affinity screening results illustrated that full-length DcGliZ showed strong and stable binding capacity with *pG, pM*, and *pN* promoters, and the response unit between the full-length DcGliZ and *pG, pM*, and *pN* promoters increased with promoter concentration until the response reached saturation status (Figure 5A). Furthermore, the kinetics screening curve indicated that the binding of full-length DcGliZ with the *pG, pM,* and *pN* promoters fitted the kinetics model, which slowly increased, steadily reached the saturation point, and then gradually decreased to the original level (Figure 5B). However, the negative control *pCP* promoter showed no relevant affinity and kinetics binding capacity with the full-length DcGliZ. The affinity and kinetics parameters calculated by the Biacore system indicated that the binding affinities of the full-length DcGliZ to the *pG, pM*, or *pN* promoter were 1.60 × 10^−7^, 1.62 × 10^−7^, or 1.04 × 1^−7^ M, respectively (Table S2), which was in accordance with the EMSA results.

## 4. Discussion

Gliotoxins and their derivatives exhibited diverse biological activities, including the suppression of macrophage immune; the inhibition of NOTCH2 transactivation; the regulation of signaling pathway; and the induction of the apoptosis in colorectal cancer cells, cervical cancer cells, chondrosarcoma cells, and chronic lymphocytic leukaemia (CLL) cells [24,40,41,42,43,44]. Hence, gliotoxin derivatives possess therapeutic potential for the prevention or treatment of cancer as well as other diseases. Gliotoxin derivatives have also been used in the clinical trial for the treatment of lung cell cancer. The combination of Illumina HiSeq and Pacific Biosciences RS sequencing platforms provides sufficient coverage depth for the comprehensive analysis of *D. cejpii* FS110 genome sequence, thus promoting the exploitation of the new bioactive compounds and their corresponding biosynthetic gene clusters from deep-sea fungi. The elucidation of the regulation mechanism of gliotoxin biosynthesis in *D. cejpii* FS110 can facilitate the exploration of new gliotoxin derivatives by approaches including transcriptional regulation and gene sequences deletion.

Transcriptional factor GliZ is encoded inside the gliotoxin biosynthetic cluster and primarily serves as a positive regulator for the gliotoxin biosynthesis in *A. fumigatus* [21]. In this study, a new GliZ transcriptional factor in *D*. *cejpii* FS110 was found by gene annotation, and DcGliZ showed a low identity of 44% with that in *A. fumigatus* by protein alignment and phylogenetic tree construction, suggesting that DcGliZ is an important pathway-specific regulator for the biosyntheis of gliotoxins and their derivatives. The core Zn_2_Cys_6_ domain of DcGliZ transcriptional factor is uniquely found in fungi, and this DcGliZ can specifically recognize and bind as homodimers to palindromic sequence motifs for the regulation of gene transcriptional level [23,45,46]. Thus, the 3D structure of Zn_2_Cys_6_ was modeled using SWISS-MODEL and PyMol for understanding the binding mechanism. Although the function of GliZ has been identified by the deletion or overexpression of *gliZ* gene for the mediation of gliotoxin production in vivo [21], the specific binding site in the promoter region of the gli cluster genes remains obscure. Previous studies implied that GliZ can bind to the promoter regions of the genes in the gli cluster, except *gliZ* and *gliA*, which encoded an efflux pump. However, the in vitro function and detailed binding affinities of GliZ protein with different promoters have not been assayed until now [22]. To determine the detailed promoters that bind to DcGliZ with strong binding affinity, thereby illustrating the in vitro regulatory mechanism of DcGliZ for gliotoxins biosyntheis, the EMSA and SPR assays were performed to identify the binding affinities of DcGliZ with different promoters for gliotoxin biosynthesis.

The full-length DcGliZ transcriptional factor was heterologously expressed as inclusion bodies in *E. coli* BL21 (DE3), and a feasible refolding method was proposed; the refolding efficiency was also improved based on a previous study [47]. The short promoters for gliotoxins biosynthesis-related genes in the gli cluster gene were cloned, biotin-labeled, and then incubated with a full-length DcGliZ transcriptional factor to perform the EMSA analysis, thus verifying the binding capacity of DcGliZ with different promoters. The retardation of the target band was visualized. The result illustrated that only *pG*, *pM*, and *pN* can combine with a full-length DcGliZ transcriptional factor, suggesting that DcGliZ has a relatively strong capability to bind with *pG*, *pM*, and *pN* promoters and regulates the biosynthesis of gliotoxin in *D*. *cejpii* FS110. The important role of the Zn_2_Cys_6_ domain in DcGliZ is confirmed by EMSA assays of heterologously expressed DcGliZ core protein with promoters *pG, pM*, and *pN*. The retardation band was observed in DcGliZ core protein–*pG* complex, however, no such retardation band was observed in DcGliZ core protein–*pM* complex and DcGliZ core protein–*pN* complex. The results indicated that the core protein of DcGliZ containing Zn_2_Cys_6_ can bind to G1-1 and G1-3 sequences of the *pG* promoter, and we assumed that other regions except the Zn_2_Cys_6_ domain probably bind to *pM* and *pN* promoters. However, the binding capacity of DcGliZ core protein with the *pG* promoter was indeed verified by EMSA assay. The *pG* promoter was obtained using genome walking kit to amplify the upstream sequences of the *gliG* gene, which encodes glutathione S-transferase (GST), and the function of GST in gliotoxin biosynthesis has been proven in our previous study [27]. The transcriptional activity of *pG* promoter was also confirmed by assaying fluorescence intensity [48]. The above results indicated that *pG* promoter plays an important role in the biosynthesis of gliotoxins and their derivatives. Further, the EMSA results demonstrated the important role of the Zn_2_Cys_6_ domain in DcGliZ protein and the function of DcGliZ in the regulation of gliotoxin biosynthesis in *D. cejpii,* and the EMSA assay of full-length DcGliZ and DcGliZ core protein also implied the possible special regulatory mechanism for gliotoxins biosynthesis in *D. cejpii* FS110 compared with those of other known GliZ transcriptional factors, which may be because of the extreme deep-sea environment in which *D*. *cejpii* FS110 lives. More importantly, the deficiency of post-translational protein processing and specific modification mechanism in *E. coli*, such as glycosylation, methylation, and acetylation, may also result in the alternation of DcGliZ core protein configuration and low binding ability of DcGliZ core protein to *pM* and *pN* promoters.

The affinity and kinetics screening between full-length DcGliZ and *pG*, *pM*, *pN*, and *pCP* were assayed by Biacore T200 biosensor. SPR assay has become an important analytical technology for the investigation on the interaction of biomolecules. Ligand can be immobilized on the SPR chip, and the targets can be specifically recognized and captured by interaction. Thus, real-time and target-mass dependent signal can be detected by monitoring changes in the refractive index (RI) [49]. This SPR technology can rapidly achieve stable analysis for the interaction of chemicals, proteins, and nucleotides with high specificity and minimal matrix effect [50]. The SPR analysis results showed that almost no binding affinity was observed between the negative control *pCP* and full-length DcGliZ (Figure 5). On the contrary, *pG*, *pM*, and *pN* showed relatively strong affinity with the full-length DcGliZ, and the kinetics curves of *pG*, *pM*, and *pN* steadily reached saturation points and then gradually decreased to their original levels. The dissociation constant (K_D_) calculated by the Biacore-T200 biosensor illustrated that the binding affinities of *pN*, *pG*, and *pM* with full-length DcGliZ were gradually decreased, whereas the K_D_ values of *pN*, *pG*, and *pM* were very close. This result is consistent with the results of EMSA.

This study was the first report on the interaction of the novel transcriptional factor DcGliZ with the promoters for the gliotoxins biosynthetic genes within the gli cluster in vitro through EMSA and SPR assays, thereby laying the theoretical foundation for the investigation of other transcriptional factors in vitro.

## 5. Conclusions

The whole genome of *D*. *cejpii* FS110 was firstly sequenced through Illumina and Pacific Biosciences sequencing, and the gliotoxin biosynthesis cluster and its relevant gene promoters were predicted and analyzed. The feature of a novel Zn_2_Cys_6_ type of transcriptional factor DcGliZ was illustrated by constructing a 3D model and phylogenetic tree. A new effective refolding strategy for inclusion bodies was developed to investigate the function of full-length DcGliZ in vitro. The EMSA and Biacore SPR assays results suggested that the full-length DcGliZ protein showed relatively strong binding capacity and stable dynamic binding level with *pG, pM,* and *pN* promoters. The DcGliZ core protein containing the Zn_2_Cy_6_ domain exhibited relatively strong binding affinity with the *pG* promoter compared with *pG* and *pN* promoters. The above results provide a promising platform for the investigation of the regulation mechanism of the *gli* cluster in different fungi, thus broadening the application prospects of gliotoxins and other marine-derived bioactive compounds in the biomedical industry.

## Supplementary Materials

The following are available online at https://www.mdpi.com/2218-273X/10/1/56/s1, Figure S1. Prediction of transcriptional factor DcGliZ regulatory region and the putative 3D structure using the programs of Pfam, SWISS-MODEL, and PyMol; Table S1. The primers and promoter sequences; Table S2. The kinetics parameter between DcGliZ and *pG*, *pM*, *pN*, and *pCP*.
