# Supplementary material for: Interaction of a Novel Zn2Cys6 Transcription Factor DcGliZ with Promoters in the Gliotoxin Biosynthetic Gene Cluster of the Deep-Sea-Derived Fungus Dichotomomyces cejpii
Source: Biomolecules. 2019 Dec 29;10(1):56. doi: 10.3390/biom10010056 (PMC7022936; doi:10.3390/biom10010056)
Supplement: Supplementary file 1 [file biomolecules-10-00056-s001.pdf]

Supplementary Materials

Interaction of a novel Zn2Cys6 transcription factor DcGliZ with promoters in the gliotoxin biosynthetic gene cluster of the deep- sea-derived fungus *Dichotomomyces cejpii*

Zi-Lei Huang, Wei Ye*, Mu-Zi Zhu, Ya-Li Kong, Sai-Ni Li, Shan Liu and Wei-Min Zhang*

[1] State Key Laboratory of Applied Microbiology Southern China, Guangdong Provincial Key Laboratory of Microbial Culture Collection and Application, Guangdong Open Laboratory of Applied Microbiology, Guangdong Institute of Microbiology,Guangdong Academy of Sciences, Guangzhou 510070, China; huangzilei15@mails. ucas.ac.cn (Z.-L.H.); zhumuzi@foxmail.com (M.-Z.Z.); 13610201546@163.com (Y.-L.K.); lisn@gdim.cn (S.-N.L.); leeshuai0305@gmail.com (S.L); 17139637739@139.com (Y.-F.C.)

* Correspondence: yewei@gdim.cn (W.Y.); wmzhang@gdim.cn (W.-M.Z.); Tel.: +86-20-87688309

Supplementary Figures

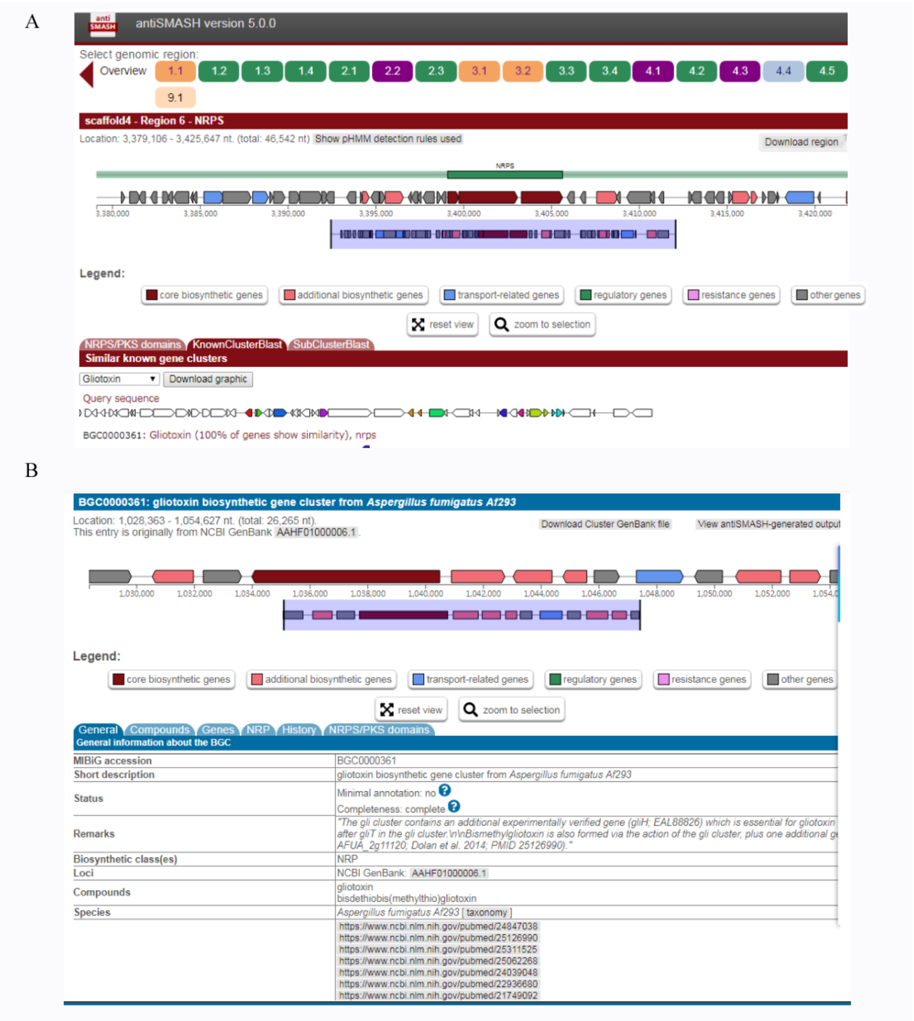

Fig. S1 The prediction of gliotoxin biosynthesis gene cluster of *D. cejpii* and the similarity with other *gli* cluster: A) the prediction of *gli* cluster of *D. cejpii* by antiSMASH 5.0.0; B) the annotation of gliotoxin biosynthetic cluster in *A. fumigutas* Af293.

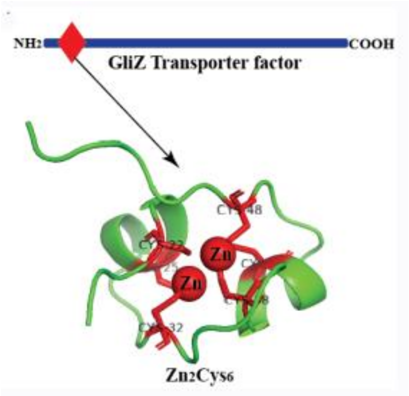

Fig.S2. Prediction of transcriptional factor DcGliZ regulatory region and the putative 3-D structure using the programs of Pfam, SWISS-MODEL and PyMol.

Table S1 The primers used in this study

Primer	Sequence
DcgliZ(core)-F	TGTTTAACTTTAAGAAGGAGATATACAATGTCGCCATTGTCCGATTCCCAG
DcgliZ(core)-R	CAGTGGTGGTGGTGGTGGTGGGTGTCGCTTATTGTGGTGTCGG
DcgliZ-F	TGTTTAACTTTAAGAAGGAGATATACAATGTCGCCATTGTCCGATTCCCAG
DcgliZ-R	AGTGGTGGTGGTGGTGGTGGAGCAGATTACAGAGCCTGCTCTGCAG
pET22b-F	TGTATATCTCCTTCTTAAAGTTAAACAAAAT
pET22b-R	CACCACCACCACCACCACTGAGATCC
pG-F	GTTTCTATGTCTATATCGGAGG
pG-R	CGAAGAGAACCAGCCTGTCC
pI-F	ATTATGCGTTTGTATATTTGAGGC
pI-R	CTGAGTCTCCTCTATTGTGGTC

pM-F	TAGTCTATCACTGAACAGTTGAATG
pM-R	TGTGATCGAGCTCAACTGATTG
pN-F	TTACTTCTTGGTCTACTCCGTATT
pN-R	GATGATGTTTAGTTGATTTTGCTG
pCP-F	TGATTTGGGCCGTCTGGCTG
pCP-R	GATTGAGATGAGGAACAGAGACG
pTF-F	GTTTGGGAAATTCTAACAAGAGTG
pTF-R	GGTATCCTGTTGCCACCTG
pA-F	CTTGACACCAGCAGAGGAGAG
pA-R	ATGCCCTGGTTACTGGATTG

TablTable S2. The kinetics parameter between DcGliZ and pG, pM, pN, pCP

Sample	ka(1/Ms)	kd(1/s)	K_D(M)	Rmax(RU)	$Chi^2(RU^2)$	Ligand	Model
pG	8.30E+04	1.32E-02	1.60E-07	16.5	6.87	DcGliZ	1:1 binding
pM	7.43E+04	1.21E-02	1.62E-07	12.9	4.24	DcGliZ	1:1 binding
pN	8.99E+04	9.39E-03	1.04E-07	13.0	7.27	DcGliZ	1:1 binding
pCP	1.02E+07	1.68E-02	1.65E-09	153.6	4.44E+05	DcGliZ	1:1 binding

ka: association rate constant, kd: dissociation rate constant

pG promoter sequence:

GTTTCTATGTCTATATCGGAGGAGTGATCAACGGCGTCCGAAAATTCGGCCCGCGGCTGT

CTTAGGGCTGGCCTGCAGGTGTAGGGTTCATTATGAAAGACTGGTAGTATTATGCTGCTA

TTTAGTCAAAAGCAGAAGAACAGTTTGTCTTTAGACCTTGAATAGATATCTGTCAAGACA

AGAGCCAAGAAAGTCCCATCACCATGGCAGACATGACCGAACGACCTTCTGATCTCGTTG

TGGACAGGCTGGTTCTCTTCG

pI promoter sequence:

ATTATGCGTTTGTATATTTGAGGCAAGGCATACCAGAAACAGTCGTTATATATTCAGTTT

CCTTGAGATGCTACATTCGGTGGCCGAAAGGGGATATGCCGACGAAAGGATGTCGATTCT

TCTTCATCGGCCTCCGAATATGTTCGAGACTGGCATATCCCAGTTTACTTATACAGAGGA

ACCATATTTCAACCCTCTAGCTGAATCTGCCTTGTCTACCTTCCCAAATCCAGGTGGATT

GATCTTGCCAGTTTATGACCACAATAGAGGAGACTCAG

pM promoter sequence:
TAGTCTATCACTGAACAGTTGAATGAATTTGTTTCAAGACAGCTAAAGCTCCATTCTAGC

CGTACGTACTGGTAGTTCTCCGCATAGAAACATATATCTCTCTGCTTGGTCAAAGTCAGG

TATCGCCGACAAGTCTTAATATCTGCAGAGTATTCGGTGACCGAAATAGGGTCGGAATAT

AAGCCATATATATCACTGTTCTGGAAATCAAGAAAATATAACAGTTGAATACTCACTTTT

CTAACATTCCAACAATCAGTTGAGCTCGATCACA

pN promoter sequence:
TTACTTCTTGGTCTACTCCGTATTGTGAATCGAAATGAGATAGAAGTATAGTTCTACTGT

ACTGGTTTCTATCTGCTTAAAATAAATACAATTGTGCCTTCTATACAGATCAGTATGTAG

AATGAACTGATAGACCTCCAGGAGGTTCTTCCTCCGAGCCTGCAACGCCCAAGAGGAGAA

CTGTGCATACGCCAGGACAGATTTTCGGCCTCCGAAAAGGACTGAGGATAGTATCTGCCA

TGTTTATATATAACTGAGGGAATAGACCAGGTAGATAACAGCAAAATCAACTAAACATCA

TC

pCP promoter sequence:
TGATTTGGGCCGTCTGGCTGTAAGACAACAGGCAAACACATCTGATGTGGTGTCCGGGGA

GGACGGCATATGAAGCATCCGAATTCCTCATATCAAAAGATCCTGTATTCAACAATCAGA

CCACCATGTCTACTTCCTCCCGTCCTATCTGATCGTCGTCTCTGTTCCTCATCTCAATC

pTF promoter sequence:
GTTTGGGAAATTCTAACAAGAGTGGAAGAACGAAAGGAATATTTACTTTGTTAAGATAGA

CCAGCAAACTATCTATTTATTAAGAACCACTCGGGGACTTTCCCCCTCGGCAGCCGAAAA

AAGTGTCCGCGGCGAGATTGTGGATCTGGCATGATCATTTATACAAATGGATAATTCCAT

GAGCTATTAATAAAGATGCAGGTATATTGTTTTCTGAAAGCATAAAAGACAGCCGAAGTA

TACAGACAACAGGCAGGTGGCAACAGGATACC

pA promoter sequence:
CTTGACACCAGCAGAGGAGAGAAAAGAAAGCAGATGTGGATATGTTTCAATCAGGGGTTG

AGTAGTTGGAATTCTGAGGGTAGAAGAAGGAAAAGATGTTGATCAGGGAGGCATTTGTAT

GTTTCCTTGTCGGAGACATGGAGACATATACACATAACAGGTAATAGGATATTGGTTAAT

GCAGCCTTCTAGACCAGGATCACTTGTCAGCAAGATGGCCTGGCCTCTGATCAGCCTATT

CATTGTTTCTGAGGGCAGTATAAGAATACTTGACCAACTGTGCTTTGTGTACAATCCAGT

AACCAGGGCAT
